# Supplementary material for: Metagenomic next-generation sequencing for the clinical diagnosis and prognosis of acute respiratory distress syndrome caused by severe pneumonia: a retrospective study
Source: PeerJ. 2020 Jul 29;8:e9623. doi: 10.7717/peerj.9623 (PMC7395598; doi:10.7717/peerj.9623)
Supplement: Supplemental Information 2 — Cox univariate analysis was performed on all factors and Cox multivariate analysis was performed with variates which were P < 0.2 of the Cox univariate analysis. [file peerj-08-9623-s002.docx]

Supplemental table Cox univariate analysis of two groups of patients

|  | coef | exp(coef) | se(coef) | z | *P*-value |
| --- | --- | --- | --- | --- | --- |
| NGS (yes/no) | -0.9585 | 0.3835 | 0.3872 | -2.475 | **0.013*** |
| Age (yr) | 0.02023 | 1.02044 | 0.01063 | 1.904 | **0.057*** |
| Sex (male/female) | 0.09157 | 1.0959 | 0.3742 | 0.245 | 0.807 |
| Length of stay in ICU (d) | -0.06728 | 0.93493 | 0.03017 | -2.23 | **0.026*** |
| Duration of mechanical ventilation (h) | -0.000662 | 0.9993385 | 0.0010875 | -0.608 | 0.543 |
| APACHE Ⅱ score before treatment | 0.10713 | 1.11308 | 0.03087 | 3.471 | **＜0.001*** |
| SOFA score before treatment | 0.16765 | 1.18252 | 0.05409 | 3.099 | **0.002*** |
| Hypertension (yes/no) | -0.1145 | 0.8918 | 0.3743 | -0.306 | 0.760 |
| Coronary heart disease (yes/no) | 0.8483 | 2.3357 | 0.4834 | 1.755 | **0.079*** |
| COPD (yes/no) | -0.1003 | 0.9045 | 0.3868 | -0.259 | 0.795 |
| Bronchiectasis (yes/no) | 0.7603 | 2.1389 | 0.5321 | 1.429 | **0.153*** |
| Chronic nephrosis (yes/no) | -0.2128 | 0.8083 | 0.5315 | -0.4 | 0.689 |
| Diabetes (yes/no) | -0.3358 | 0.7148 | 0.5313 | -0.632 | 0.527 |
| Immunosuppression (yes/no) | 0.3641 | 1.4392 | 0.3742 | 0.973 | 0.331 |
| Tumor (yes/no) | 0.3259 | 1.3853 | 0.3744 | 0.871 | 0.384 |
| Smoking (yes/no) | 0.06306 | 1.06509 | 0.34507 | 0.183 | 0.855 |
| Drinking (yes/no) | 0.3113 | 1.3652 | 0.5314 | 0.586 | 0.558 |
| PCT（ug/L） | -0.006918 | 0.993106 | 0.008162 | -0.848 | 0.397 |
| WBC（10^9^/L） | 0.02517 | 1.02549 | 0.02384 | 1.056 | 0.291 |
| Hb（g/L） | -0.014806 | 0.985303 | 0.006503 | -2.277 | **0.023*** |
| PLT（10^9^/L） | -0.002613 | 0.997391 | 0.002214 | -1.18 | 0.238 |
| Cr（umol/L） | -4.68E-05 | 1 | 0.0007256 | -0.065 | 0.949 |
| T.Bil（mmol/L） | 0.007238 | 1.007264 | 0.004939 | 1.465 | **0.143*** |
| ALT（IU/L） | -0.0013 | 0.998701 | 0.001818 | -0.715 | 0.475 |
| Alb（g/L） | 0.02717 | 1.02754 | 0.0305 | 0.891 | 0.373 |
| APTT（sec） | 0.001949 | 1.001951 | 0.010613 | 0.184 | 0.854 |
| BNP（pg/ml） | -1.06E-05 | 1 | 4.105E-05 | -0.259 | 0.796 |
| Lac（mmol/L） | -0.1029 | 0.9022 | 0.127 | -0.81 | 0.418 |
| PH | 1.28 | 3.598 | 1.995 | 0.642 | 0.521 |
| PaO2 | 0.0001771 | 1.0001771 | 0.0057154 | 0.031 | 0.975 |
| PaCO2 | 0.009267 | 1.009311 | 0.009976 | 0.929 | 0.353 |
| Be | 0.03927 | 1.04005 | 0.02537 | 1.548 | **0.122*** |
| OI | -0.001195 | 0.998805 | 0.002628 | -0.455 | 0.649 |
| FiO2 | 0.6049 | 1.831 | 0.8365 | 0.723 | 0.470 |
| Peep | 0.02499 | 1.02531 | 0.04861 | 0.514 | 0.607 |
| Use of vasoactive agent (yes/no) | 0.4582 | 1.5812 | 0.3562 | 1.286 | **0.198*** |
| CRRT (yes/no) | 0.4549 | 1.5761 | 0.4026 | 1.13 | 0.259 |
| ECMO (yes/no) | -1.4414 | 0.2366 | 1.0147 | -1.42 | **0.155*** |
| Prone positioning (yes/no) | 0.3034 | 1.3545 | 0.3744 | 0.81 | 0.418 |
| ICU cost (CNY) | -3.69E-06 | 1 | 2.228E-06 | -1.655 | **0.098*** |

Note: Cox univariate analysis was performed on all factors and Cox multivariate analysis was performed with variates which were *P* < 0.2 of the Cox univariate analysis.
